# Complete Blood Count-Derived Inflammatory Indices in Catatonia: A Retrospective Matched Case–Control Study

**DOI:** 10.3390/diagnostics16132110

**Published:** 2026-07-06

**Authors:** Octavia Căpățînă, Adela Hanga, Sonia Tivadar, Andrei Hopulele-Petri, Denis Paval, Mihaela Fadgyas Stanculete

**Affiliations:** 1 Department of Neuroscience, Iuliu Hațieganu University of Medicine and Pharmacy, 400347 Cluj-Napoca, Romaniamihaelastanculete@yahoo.com (M.F.S.); 2 County Emergency Hospital Cluj-Napoca, 400347 Cluj-Napoca, Romania; 3 “Dr. Constantin Papilian” Emergency Military Clinical Hospital, 22 General Traian Moșoiu Street, 400132 Cluj-Napoca, Romania

**Keywords:** catatonia, complete blood count, systemic immune–inflammation index, systemic inflammation response index, inflammatory markers, psychiatric inpatients, diagnosis, screening

## Abstract

**Background/Objectives**: Catatonia is a severe transdiagnostic neuropsychiatric syndrome for which accessible biological correlates remain insufficiently characterized. This study explored whether complete blood count (CBC)-derived inflammatory indices differ between psychiatric inpatients with catatonia and matched psychiatric controls without catatonia. **Methods**: This retrospective matched case–control study included 46 patients with catatonia and 46 psychiatric controls selected from the same clinical setting and study period. Controls were frequency-matched by sex, age distribution, and broad psychiatric diagnosis. CBC parameters obtained within the first 24 h of admission were used to calculate the neutrophil-to-lymphocyte ratio (NLR), platelet-to-lymphocyte ratio (PLR), monocyte-to-lymphocyte ratio (MLR), systemic immune–inflammation index (SII), and systemic inflammation response index (SIRI). Group comparisons, adjusted log–linear regression models, Spearman correlations with documented catatonic signs, and exploratory receiver operating characteristic analyses were performed. **Results**: SII was higher in patients with catatonia than in controls and remained significant after Bonferroni correction (median 584 [IQR 468–823] vs. 476 [IQR 339–619], Bonferroni-adjusted *p* = 0.032). In secondary adjusted models, catatonia was associated with higher SII and SIRI after adjustment for body mass index, smoking, antipsychotic exposure, diabetes mellitus, and arterial hypertension. No inflammatory index correlated significantly with the number of documented catatonic signs after correction. Exploratory discrimination was poor to fair, with SII showing the highest AUC (0.665, 95% CI 0.550–0.773). **Conclusions**: CBC-derived indices, particularly SII, may reflect systemic inflammatory or physiological stress burden in catatonia, but they should be interpreted as exploratory markers rather than diagnostic biomarkers.

## 1. Introduction

Catatonia is a severe neuropsychiatric syndrome characterized by disturbances in motor activity, speech, behavior, affect, and autonomic regulation. Although historically conceptualized as a subtype of schizophrenia, contemporary diagnostic systems define catatonia as a transdiagnostic syndrome that may occur in the context of psychotic disorders, mood disorders, neurological diseases, general medical conditions, substance use, or medication-related states [1,2]. Its clinical presentation is heterogeneous and may include hypokinetic signs such as stupor, mutism, negativism, rigidity, and posturing, as well as hyperkinetic or parakinetic manifestations such as agitation, stereotypies, mannerisms, grimacing, and echophenomena [1,3]. This heterogeneity contributes to frequent under-recognition, delayed diagnosis, and potential exposure to inappropriate treatment strategies.

Early identification of catatonia is clinically important because the syndrome is associated with substantial morbidity and may progress to severe medical complications, including dehydration, malnutrition, pressure ulcers, infections, venous thromboembolism, autonomic instability, and malignant catatonia. Despite this risk, catatonia is often underdiagnosed in both psychiatric and general medical settings. Recent evidence suggests that clinicians tend to recognize classical hypokinetic presentations more readily, while movement-rich or parakinetic forms may be overlooked [4]. The absence of a specific diagnostic biomarker further reinforces the need for objective, accessible biological correlates that may complement clinical assessment and improve understanding of catatonia pathophysiology.

The mechanisms underlying catatonia remain incompletely understood. Current models implicate dysfunction in GABAergic, dopaminergic, and glutamatergic neurotransmission, particularly within cortico-striato-thalamo-cortical circuits involved in motivation, movement initiation, and behavioral regulation [5,6]. In parallel, increasing attention has been given to the possible role of immune dysregulation and neuroinflammation. Catatonia has been described in association with infections, autoimmune encephalitis, systemic inflammatory diseases, neuroleptic malignant syndrome, and other immune-mediated conditions [6]. Among autoimmune causes, N-methyl-D-aspartate receptor encephalitis is particularly relevant, as it can reproduce the full spectrum of catatonic signs and supports the hypothesis that glutamatergic hypofunction may contribute to catatonic states [6].

The relationship between catatonia and inflammation is likely complex. Activation of the innate immune system may produce “sickness behavior,” a behavioral state characterized by motor slowing, withdrawal, reduced oral intake, fatigue, and decreased social interaction, which partially overlaps with the neurovegetative and psychomotor features of catatonia [6]. However, evidence for acute-phase systemic inflammation in catatonia remains limited and inconsistent. Previous studies have examined markers such as white blood cell count, C-reactive protein, serum iron, creatine kinase, and D-dimer, with heterogeneous findings [6]. This suggests that simple isolated inflammatory markers may not fully capture the biological changes associated with catatonia, and that composite indices reflecting the balance between different immune-cell populations may provide additional information.

Complete blood count-derived inflammatory indices have emerged as inexpensive and readily available markers of systemic immune activation. The neutrophil-to-lymphocyte ratio (NLR) reflects the relationship between innate immune activation and adaptive immune regulation and has been reported to be elevated across several psychiatric disorders, including schizophrenia, bipolar disorder, and major depressive disorder [7]. Other indices, such as the platelet-to-lymphocyte ratio (PLR), monocyte-to-lymphocyte ratio (MLR), systemic immune–inflammation index (SII), and systemic inflammation response index (SIRI), integrate additional inflammatory components, including platelets and monocytes, and may reflect broader immune and inflammatory processes [8]. Although these markers are non-specific and influenced by several clinical factors, their low cost, availability, and feasibility in retrospective datasets make them attractive tools for exploratory psychiatric research.

Evidence regarding CBC-derived inflammatory indices in catatonia remains scarce. To date, the most directly relevant study is that of Şahin et al. [9], who compared patients with catatonia due to mental disorders with healthy controls and found significantly higher NLR values in the catatonia group, while PLR and MLR did not differ significantly. That study supported the hypothesis of low-grade systemic inflammation in catatonia, but its use of healthy controls made it difficult to determine whether the inflammatory signal was specific to catatonia or reflected severe psychiatric illness more broadly. This distinction is important because inflammatory markers are frequently altered across psychiatric diagnoses, including psychosis and mood disorders, both of which may themselves be associated with catatonia [7,10,11].

The present study was designed to further investigate CBC-derived inflammatory indices in catatonia using a matched psychiatric control group. Unlike comparisons with healthy controls, psychiatric controls allow a more specific examination of whether inflammatory indices differ between patients with catatonia and patients with similar psychiatric diagnoses but without catatonia. The study focused on five primary CBC-derived inflammatory markers: NLR, PLR, MLR, SII, and SIRI. Given the potential influence of demographic, lifestyle, metabolic, cardiovascular, and treatment-related factors on inflammatory indices, the analysis also considered relevant confounders, including body mass index, smoking status, glycemic/metabolic variables, arterial hypertension, and exposure to antipsychotic medication.

The aim of this study was therefore to explore whether CBC-derived inflammatory indices differ between psychiatric inpatients with catatonia and matched psychiatric controls without catatonia. A secondary objective was to examine whether these indices are associated with the number of documented catatonic signs and to describe, in an exploratory manner, their ability to discriminate between catatonic and non-catatonic psychiatric presentations. Given the scarcity of previous data, analyses of individual CBC-derived markers were considered exploratory and hypothesis-generating.

We hypothesized that catatonia would be associated with higher CBC-derived inflammatory indices, particularly composite indices incorporating neutrophil, lymphocyte, monocyte, and platelet parameters. We further explored whether any observed associations remained present after adjustment for BMI, smoking status, and antipsychotic exposure. As a secondary exploratory hypothesis, we examined whether higher values of NLR, PLR, MLR, SII, and SIRI were associated with a greater number of documented catatonic signs.

## 2. Materials and Methods

### 2.1. Study Design and Setting

This was a retrospective matched case–control study conducted at The Psychiatric Clinic in Cluj-Napoca, Romania. Patients were collected from hospital records over a five-year period, between July 2019 and July 2024. The study included two groups: patients diagnosed with catatonia and psychiatric control patients without catatonia. The final database included 46 patients with catatonia and 46 control patients without catatonia. The study was approved by the Ethics Committee of Cluj-Napoca Emergency County Hospital (approval no. 31911/7 August 2024).

### 2.2. Study Population

#### 2.2.1. Case Group

The case group consisted of all psychiatric inpatients diagnosed with catatonia at admission to the hospital in the study period. Catatonia symptoms were identified based on clinical documentation and the presence of catatonic signs recorded in the medical files. The catatonic signs, as described by the ICD-11 for the diagnosis of catatonia, were assessed when available: staring, ambitendency, negativism, stupor, mutism, hyperactivity, grimacing, mannerisms, posturing, stereotypies, rigidity, echophenomena, verbigeration, waxy flexibility and catalepsy, in order to measure the severity of catatonia. Two investigators independently reviewed the clinical documentation to confirm fulfillment of the ICD-11 diagnostic criteria for catatonia and coded each of the 15 ICD-11 catatonic signs as present or absent. Both reviewers confirmed that all included patients met the ICD-11 diagnostic threshold, corresponding to 100% agreement regarding case classification. Cohen’s kappa was not estimated for the binary diagnostic classification because all ratings were positive and therefore lacked category variability. Agreement regarding the total number of documented catatonic signs was assessed using a two-way random-effects intraclass correlation coefficient for absolute agreement and single measurements, ICC (2,1), and was excellent (ICC = 0.989). The reviewers recorded the same total number of signs in 39 of 46 cases (84.8%); all remaining ratings differed by only one sign. Symptom-level discrepancies were reviewed by a third investigator and resolved by consensus.

#### 2.2.2. Control Group

The control group consisted of psychiatric inpatients without catatonia, selected from the same clinical setting and study period. Controls were selected at a 1:1 ratio from psychiatric inpatients without catatonia. Frequency matching was performed at the group level according to sex, age distribution, and broad psychiatric diagnosis categories: schizophrenia, acute psychotic episode, bipolar disorder, and schizoaffective disorder. Because matching was performed at the frequency rather than the individual-pair level, analyses were conducted as independent-group comparisons. Controls were selected consecutively in chronological order according to admission date, within strata defined by broad psychiatric diagnosis, to approximate the age and sex distribution of the catatonia group. The same exclusion criteria were applied to both groups, and control selection was performed without reference to complete blood count values or derived inflammatory indices.

#### 2.2.3. Exclusion Criteria

Patients were excluded if they had conditions or treatments that could substantially influence inflammatory markers derived from the complete blood count. The exclusion criteria were: catatonia due to an organic disorder, acute infections, pregnancy, malignancy, current treatment with corticosteroids and current treatment with anti-inflammatory drugs.

### 2.3. Variables Collected

The following variables were extracted from the medical records: Demographic and clinical variables: age, sex, psychiatric diagnosis, duration of hospitalization, number of documented catatonic signs, and drug-naïve versus exposed to antipsychotic medication; Anthropometric and lifestyle variables: body mass index (BMI), smoking status; Metabolic and cardiovascular variables: total cholesterol, triglycerides, HDL cholesterol, LDL cholesterol, diagnosis of arterial hypertension and diagnosis of diabetes. These variables were considered as potential confounders or exploratory covariates because metabolic and cardiovascular factors may influence systemic inflammatory status. Complete blood count parameters were collected from blood samples obtained within the first 24 h of admission. The following hematological values were included: leukocyte count, neutrophil count, lymphocyte count, monocyte count, and platelet count. Based on these values, the following inflammatory markers were calculated: neutrophil-to-lymphocyte ratio, NLR; platelet-to-lymphocyte ratio, PLR; monocyte-to-lymphocyte ratio, MLR; systemic immune–inflammation index, SII = platelets × neutrophils/lymphocytes; systemic inflammation response index, SIRI = neutrophils × monocytes/lymphocytes. The primary inflammatory markers of interest were NLR, PLR, MLR, SII and SIRI. Blood samples were collected within the first 24 h after admission in order to reduce the influence of hospitalization-related factors and treatment changes on inflammatory parameters.

### 2.4. Statistical Analysis

No a priori sample size calculation was performed because this retrospective study included all eligible patients with a documented diagnosis of catatonia admitted during the predefined study period. The number of cases was therefore determined by the complete available cohort, and controls were selected at a 1:1 ratio. A sensitivity analysis based on the final sample of 46 patients per group indicated that, assuming a two-sided independent-group comparison and 80% power, the minimum detectable standardized effect was approximately Cohen’s d = 0.59 at α = 0.05 and d = 0.73 at the Bonferroni-adjusted α = 0.01. Continuous variables were tested for normality using the Shapiro–Wilk test and visual inspection of distributions. Normally distributed variables were expressed as mean ± standard deviation, while non-normally distributed variables were expressed as median and interquartile range. Categorical variables were expressed as frequencies and percentages. Comparisons between patients with catatonia and controls were performed using the Mann–Whitney U test or independent samples *t*-test for continuous variables, and the chi-square test or Fisher’s exact test for categorical variables.

The primary inflammatory markers were NLR, PLR, MLR, SII and SIRI. Because inflammatory markers showed skewed distributions, unadjusted comparisons were performed using the Mann–Whitney U test with correction for multiple comparisons across the five primary indices. These five indices were treated as a single family of primary comparisons and were therefore subject to Bonferroni correction. Individual leukocyte, neutrophil, lymphocyte, monocyte, and platelet counts were examined only as exploratory component-level variables to facilitate interpretation of the composite indices; their *p* values were reported without adjustment for multiple comparisons. Secondary adjusted analyses were performed using linear regression models with log-transformed inflammatory markers as dependent variables. These models were used to explore whether observed group differences persisted after adjustment for potential confounders and were not interpreted as replacing the corrected unadjusted comparisons. The main adjusted models included catatonia status as the independent variable of interest and were adjusted for BMI and smoking status. Secondary sensitivity analyses included additional adjustment for antipsychotic exposure, diabetes mellitus, and arterial hypertension.

In the catatonia group, the association between inflammatory markers and the number of documented catatonic signs was assessed using Spearman correlation. Because catatonic signs were retrospectively extracted from clinical documentation and not assessed prospectively using a standardized rating scale, this variable was interpreted as an approximate indicator of documented clinical features rather than a validated severity score. Receiver operating characteristic curve analysis was performed strictly as an exploratory analysis to describe discriminatory performance; the resulting cut-offs were not considered clinically validated. A *p*-value below 0.05 was considered statistically significant, with correction for multiple comparisons applied to the five primary inflammatory markers. Statistical analyses were performed using IBM SPSS Statistics, version 22.1.

## 3. Results

The study included 46 patients with catatonia and 46 psychiatric controls without catatonia. Age, sex distribution, and psychiatric diagnosis were comparable between groups, supporting the frequency-matching strategy. The demographic and clinical characteristics of the groups are presented in Table 1. Patients with catatonia had a longer hospitalization duration than controls (median 21.0 [IQR 15.0–27.8] vs. 14.5 [IQR 9.2–22.0] days, *p* = 0.003). The median number of documented catatonic signs among cases was 7.0 (4.2–8.0).

Table 2 presents the metabolic and cardiovascular variables for the two groups and shows that the metabolic risk is similar for the research populations.

Among the five primary CBC-derived inflammatory indices, SII was higher in patients with catatonia than controls and remained significant after Bonferroni correction (median 584 [IQR 468–823] vs. 476 [IQR 339–619], raw *p* = 0.006, adjusted *p* = 0.032). NLR, PLR, MLR, and SIRI did not remain significant after correction in the unadjusted comparisons. Component-level analysis showed that the SII difference was accompanied by higher platelet and neutrophil counts in the catatonia group, whereas lymphocyte counts did not differ between groups. The results of the analysis are presented in Table 3.

Table 4 presents the results of secondary adjusted log–linear regression models for the primary inflammatory indices: Model 1 adjusted for BMI and smoking; Model 2 adjusted for Model 1 covariates plus antipsychotic exposure; Model 3 adjusted for Model 2 covariates plus diabetes mellitus and arterial hypertension. In these secondary adjusted models, catatonia was associated with higher SII and SIRI. For SII, catatonia was associated with a 47.5% higher value (95% CI 18.3% to 84.0%, adjusted *p* = 0.004); for SIRI, catatonia was associated with a 52.9% higher value (95% CI 17.6% to 98.7%, adjusted *p* = 0.009). These associations remained after additional adjustment for antipsychotic exposure and after further adjustment for diabetes mellitus and arterial hypertension. These adjusted results should be interpreted as exploratory evidence supporting the robustness of the SII and SIRI associations, rather than as replacing the corrected unadjusted comparisons.

**Table 4:** Adjusted log–linear regression models for primary inflammatory indices.

Marker	Model	% Difference (95% CI)	Raw *p*	Bonferroni-Adjusted *p*
NLR	Model 1	29.7% (6.0 to 58.6)	0.012	0.060
NLR	Model 2	29.1% (5.5 to 58.1)	0.014	—
NLR	Model 3	27.6% (4.1 to 56.4)	0.020	0.098
PLR	Model 1	17.1% (−1.0 to 38.6)	0.066	0.328
PLR	Model 2	17.2% (−1.1 to 38.8)	0.066	—
PLR	Model 3	14.6% (−2.8 to 35.2)	0.104	0.521
MLR	Model 1	21.4% (2.8 to 43.3)	0.023	0.114
MLR	Model 2	20.6% (2.2 to 42.3)	0.027	—
MLR	Model 3	19.2% (0.8 to 41.0)	0.041	0.204
SII	Model 1	47.5% (18.3 to 84.0)	<0.001	0.004
SII	Model 2	47.7% (18.2 to 84.4)	<0.001	—
SII	Model 3	45.5% (16.7 to 81.4)	0.001	0.005
SIRI	Model 1	52.9% (17.6 to 98.7)	0.002	0.009
SIRI	Model 2	51.9% (16.8 to 97.7)	0.002	—
SIRI	Model 3	51.3% (15.7 to 97.8)	0.003	0.014

Note: Values are percent differences associated with catatonia status after back-transformation from log–linear models. Model 1: adjusted for BMI and smoking. Model 2: Model 1 + antipsychotic exposure. Model 3: Model 2 + diabetes mellitus and arterial hypertension. Bonferroni-adjusted *p* values are shown for Models 1 and 3; — indicates not reported/applicable. A forest plot was added as Figure 1 to facilitate visual comparison of the fully adjusted associations from Model 3. The figure shows the back-transformed percentage differences and 95% confidence intervals for NLR, PLR, MLR, SII, and SIRI. Consistent with the regression analysis, SII and SIRI showed the strongest adjusted associations with catatonia.

**Figure 1:**
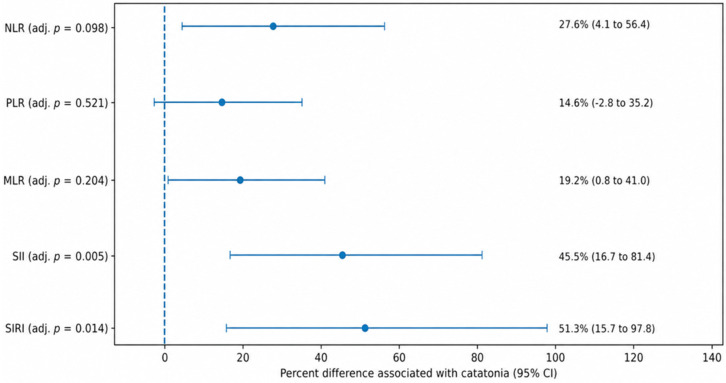
Forest plot of the fully adjusted associations between catatonia status and the five primary CBC-derived inflammatory indices. Estimates are shown as back-transformed percentage differences with 95% confidence intervals from Model 3 of Table 4, adjusted for BMI, smoking status, antipsychotic exposure, diabetes mellitus, and arterial hypertension. Positive values indicate higher marker levels in patients with catatonia relative to psychiatric controls. SII and SIRI showed the strongest adjusted associations and remained significant after correction for multiple comparisons.

No inflammatory index correlated significantly with the number of documented catatonic signs after multiple-comparison correction, as shown in Table 5.

In exploratory ROC analysis, discriminatory ability was poor to fair; SII showed the highest AUC (AUC 0.665, 95% CI 0.550–0.773). Table 6 presents the results of the analysis.

Among CBC-derived inflammatory indices, SII showed the most consistent association with catatonia, being significantly higher in patients with catatonia in unadjusted analyses and remaining significant after correction for multiple comparisons. In secondary adjusted models, both SII and SIRI were associated with catatonia status after accounting for BMI, smoking, antipsychotic exposure, diabetes mellitus, and arterial hypertension. However, NLR, PLR, MLR, and SIRI did not survive correction in the unadjusted comparisons, ROC analyses showed only poor-to-fair discrimination, and inflammatory indices were not significantly associated with the number of documented catatonic signs.

## 4. Discussion

In this retrospective matched case–control study, patients with catatonia showed higher SII values than psychiatric controls without catatonia, while the other primary CBC-derived inflammatory indices did not remain significant after correction for multiple comparisons in unadjusted analyses. In secondary adjusted models, SII and SIRI were associated with catatonia status after adjustment for BMI, smoking, antipsychotic exposure, diabetes mellitus, and arterial hypertension. These findings suggest that catatonia may be associated with a state of low-grade systemic inflammation, particularly reflected by composite indices incorporating neutrophils, lymphocytes, monocytes, and platelets, but the results should be interpreted as exploratory, because they do not establish a catatonia-specific inflammatory biomarker. Rather, they suggest that selected CBC-derived indices, particularly SII, may capture aspects of systemic inflammatory or physiological stress burden in patients presenting with catatonia.

The present findings are partly consistent with the study by Şahin et al. [9], who reported significantly higher NLR values in patients with catatonia compared with healthy controls, while PLR and MLR did not differ significantly between groups. Their study controlled for age, sex, smoking, and BMI and excluded several medical conditions that could influence inflammatory markers. In contrast, the present study did not find NLR to be the strongest marker after correction for multiple testing. Instead, only SII remained significant in the corrected unadjusted comparison, while SII and SIRI were associated with catatonia in secondary adjusted models. This difference may reflect the use of psychiatric rather than healthy controls, the different composition of the sample, or the broader cellular components included in composite indices such as SII and SIRI.

The use of psychiatric controls in the present study is an important methodological distinction. Şahin et al. [9] compared catatonia patients with healthy controls, which is useful for detecting general inflammation but makes it difficult to determine whether the observed inflammatory signal is specific to catatonia or reflects severe psychiatric illness more broadly. In the current study, controls were selected from the same psychiatric setting and matched by age, sex, and psychiatric diagnosis, reducing confounding by psychiatric morbidity. This distinction is important because CBC-derived inflammatory markers have been reported to be elevated across several severe mental disorders, including schizophrenia, bipolar disorder, and major depressive disorder [10,11,12,13].

The findings also need to be interpreted in light of evidence from psychotic disorders. Meta-analytic evidence indicates that NLR is increased in schizophrenia and that antipsychotic exposure may influence the interpretation of this marker [14]. Similarly, Mazza et al. [11] reported higher NLR and MLR in patients with non-affective psychosis compared with healthy controls, whereas PLR appeared less robust. In the present study, however, diagnosis was frequency-matched between groups, and PLR was not independently associated with catatonia. Therefore, the observed differences should not be interpreted simply as evidence that catatonia has a distinct inflammatory profile; they may also reflect differences in acute episode severity, physiological stress, immobility, or other unmeasured factors.

The relationship between inflammation and catatonia is biologically plausible but remains incompletely understood. In a large electronic health record study, Rogers et al. [15] found mixed evidence for peripheral inflammatory abnormalities in catatonia: catatonia was associated with lower serum iron and higher creatine kinase, but not consistently with higher CRP or white blood cell count. Their findings suggest that routine inflammatory markers may not uniformly identify inflammatory changes in catatonia. The present findings are compatible with this uncertainty: the association with SII may indicate inflammatory activation, but it may also reflect non-specific admission-state physiology.

Recent literature also supports a possible link between catatonia-like or psychomotor features and immune activation. Messina et al. [16] examined patients with schizophrenia and found that catatonia-like behavior, including emotional withdrawal, blunted affect, and psychomotor slowing, was associated with the lymphocyte-to-monocyte ratio. Although this study did not assess catatonia as a full syndrome, it supports the idea that psychomotor and affective-motor abnormalities may be related to peripheral immune changes. Similarly, Aslan and Demir [17] reported inflammatory differences in major depressive disorder subtypes, including patients with catatonic features. Together, these findings suggest that inflammation may be linked not only to diagnostic categories but also to transdiagnostic motor–behavioral dimensions.

The present finding that SII and SIRI were more informative than NLR, PLR, or MLR alone may be particularly important. SII combines platelet, neutrophil, and lymphocyte counts, while SIRI combines neutrophil, monocyte, and lymphocyte counts. These indices may therefore better reflect the interaction between innate immune activation, adaptive immune regulation, and platelet-related inflammatory activity. Emerging studies in schizophrenia and bipolar disorder have reported increased SII values and have suggested that composite inflammatory indices may be useful markers of systemic immune activation in severe psychiatric disorders [18,19,20]. To our knowledge, however, SII and SIRI have not been widely studied in catatonia, which makes the present findings potentially novel. The interpretation of SII deserves particular caution. SII is calculated as platelets × neutrophils/lymphocytes. In the present study, higher SII in the catatonia group appears to be mainly driven by higher platelet and neutrophil counts, whereas lymphocyte counts were similar between groups. Neutrophilia may occur in the context of innate immune activation, but it can also reflect acute physiological stress, sympathetic or hypothalamic–pituitary–adrenal axis activation, glucocorticoid-related leukocyte redistribution, dehydration, or hemoconcentration. Similarly, a higher platelet count may reflect reactive hematological or volume-related changes. Because platelet function and activation markers were not measured, the present results should not be interpreted as direct evidence of platelet activation [8]. Therefore, this finding cannot surely be interpreted as evidence of impaired adaptive immune regulation. It may also reflect a combination of innate immune activation, platelet-related activation, physiological stress, dehydration, immobility, or unmeasured medical burden. Also, it is worth noting here that smoking was numerically more frequent among controls than among patients with catatonia. Because smoking is associated with increased leukocyte and neutrophil counts, this imbalance would be expected to increase neutrophil-related indices in the control group and thus potentially attenuate, rather than exaggerate, the observed SII difference [8]. However, smoking intensity, duration, and cumulative exposure were unavailable; therefore, residual confounding by smoking could not be quantified or entirely excluded. Patients with catatonia also had longer hospital stays than controls, which may reflect greater clinical severity or a higher medical and functional burden. Length of stay was not included as a covariate because it was determined after the CBC measurement and may represent a consequence of catatonia, its complications, or the subsequent treatment course rather than a baseline confounder. Nevertheless, unmeasured factors present before admission and associated with a longer subsequent hospitalization—such as illness duration, immobility, dehydration, reduced nutritional intake, or medical deterioration—may also have influenced the admission CBC values. Similarly, although SIRI was associated with catatonia in adjusted models, it did not remain significant after correction in the unadjusted comparisons. This difference may reflect the distinct statistical estimands of the rank-based unadjusted analysis and the covariate-adjusted log–linear models. The strengthening of the association occurred after adjustment for BMI and smoking, suggesting possible negative confounding or statistical suppression, potentially related to the higher smoking prevalence among controls. Nevertheless, because the corrected unadjusted comparison was not significant and the contribution of individual covariates was not formally decomposed, the SIRI finding should be regarded as secondary and hypothesis-generating. These findings support further investigation of composite CBC-derived indices in catatonia.

The role of antipsychotic exposure also deserves attention. In the present study, antipsychotic exposure was considered in sensitivity analyses because antipsychotic treatment may influence immune cell counts and inflammatory indices. This concern is supported by previous studies in schizophrenia and psychosis, where inflammatory markers may differ according to illness stage, treatment status, and medication exposure [14,21]. The persistence of SII and SIRI associations after adjustment for binary antipsychotic exposure suggests that the observed findings are not explained solely by whether patients were drug-naïve or previously exposed. Nevertheless, this adjustment is incomplete because antipsychotic type, dose, duration, and route of administration were not included in the analysis.

The absence of a significant association between CBC-derived indices and the number of documented catatonic signs should be interpreted cautiously. Catatonic signs were retrospectively extracted from clinical files and were not assessed prospectively using a standardized instrument such as the Bush–Francis Catatonia Rating Scale. Therefore, this variable should be considered an approximate measure of documented clinical features rather than a validated catatonia severity score. Moreover, the number of documented signs may not fully capture biological severity, duration of catatonia, autonomic instability, malignant features, immobility, dehydration, or treatment delay. Similar limitations have been emphasized in retrospective studies of catatonia, where clinical identification depends heavily on the quality and consistency of medical record documentation [15].

The exploratory ROC analysis showed only poor-to-fair discriminatory performance, with SII showing the highest AUC. This indicates that CBC-derived inflammatory indices should not be interpreted as diagnostic biomarkers for catatonia. The cut-offs reported in the ROC analysis were selected statistically within the same sample and were not validated in an independent cohort. Therefore, these indices may represent accessible, low-cost markers of inflammatory burden that could complement clinical assessment and help generate hypotheses about catatonia pathophysiology. This interpretation is consistent with broader reviews of catatonia and immunity, which conclude that immune dysregulation may contribute to some cases of catatonia, but that evidence for acute-phase systemic inflammation remains heterogeneous [6].

The findings also have potential implications for treatment approaches. If inflammation plays a causal role in catatonia pathophysiology, anti-inflammatory interventions—including NSAIDs, corticosteroids, or targeted immunotherapy—might be beneficial in selected patients, particularly those with markedly elevated inflammatory markers, treatment resistance, or evidence of autoimmune mechanisms. Several case reports and small series have described successful treatment of catatonia with immunotherapy in autoimmune-mediated cases, but systematic clinical trials are lacking. These findings may also be relevant for future work on the mechanisms and treatment of catatonia. In some patients, immune or inflammatory processes may contribute to the clinical picture, especially when catatonia occurs with autoimmune features, treatment resistance, or clear inflammatory abnormalities. However, our study cannot determine whether the differences in CBC-derived indices are part of the mechanism of catatonia or whether they reflect non-specific factors such as acute stress, dehydration, immobility, or medical burden. For this reason, the results should be interpreted cautiously. Catatonia has also been linked to disturbances in neurotransmitter systems involved in motor and behavioral regulation, including GABAergic, dopaminergic, and glutamatergic pathways. From this perspective, work on glutamate-based treatment strategies in schizophrenia and related severe psychiatric disorders may provide useful background for future studies of catatonia, although it does not directly explain the hematological findings reported here [22].

This study has several limitations. First, the retrospective design limits causal inference and depends on the completeness of clinical documentation. Although blood samples were obtained within the first 24 h of admission, this timing may not eliminate admission-state confounding. Patients with catatonia may present with immobility, reduced oral intake, dehydration, physiological stress, or delayed medical deterioration before admission, all of which may influence neutrophil and platelet counts independently of catatonia-specific immune mechanisms. Second, although cases and controls were matched by age, sex, and psychiatric diagnosis, matching was performed at the frequency level rather than the individual-pair level, and residual confounding may persist. Third, smoking was available only as a binary variable, and information on cigarettes per day, duration of smoking, or pack-years was unavailable; therefore, residual confounding by smoking intensity cannot be excluded. Fourth, antipsychotic exposure was also binary, without information on antipsychotic type, dose, route, or duration. Fifth, CBC-derived inflammatory indices are non-specific and may be influenced by BMI, metabolic status, cardiovascular risk, medication exposure, unrecognized inflammatory or infectious conditions, dehydration, and immobility. Sixth, cytokines, CRP, ferritin, serum iron, and other immunological markers were not included in the main analysis, limiting mechanistic interpretation. Finally, the sample size may have limited power for subgroup analyses, particularly analyses stratified by psychiatric diagnosis or antipsychotic exposure. The relatively small sample size limited statistical sensitivity, particularly after correction for multiple comparisons. Therefore, the non-significant findings for several inflammatory indices should not be interpreted as evidence of equivalence or absence of association, as small or moderate effects may have remained undetected. These findings therefore remain vulnerable to type II error and require confirmation in larger, preferably multicenter cohorts.

## 5. Conclusions

In conclusion, this retrospective matched case–control study found that SII was higher in patients with catatonia than in psychiatric controls and remained significant after correction for multiple comparisons. In secondary adjusted models, SII and SIRI were associated with catatonia status. However, these findings should be interpreted cautiously, as the SII difference appeared to be driven mainly by higher platelet and neutrophil counts, and residual confounding by medical burden, smoking, dehydration, immobility, medication exposure, and admission-state physiology cannot be excluded. CBC-derived indices showed poor-to-fair discriminatory performance and were not associated with the number of documented catatonic signs. These results should therefore be considered exploratory and hypothesis-generating rather than evidence for diagnostic biomarkers of catatonia.

## Figures and Tables

**Table 1:** Demographic and clinical characteristics.

Variable	Catatonia (*n* = 46)	Control (*n* = 46)	*p* Value
Age, years	36.0 (26.0–54.8)	34.0 (27.0–46.0)	0.407
Length of hospitalization, days	21.0 (15.0–27.8)	14.5 (9.2–22.0)	0.003
Number of documented catatonic signs (cases only)	7.0 (4.2–8.0)	N/A	
Sex: Female	34/46 (73.9%)	36/46 (78.3%)	0.807
Sex: Male	12/46 (26.1%)	10/46 (21.7%)	
Diagnosis: Schizophrenia	26/46 (56.5%)	28/46 (60.9%)	0.973
Diagnosis: Acute psychotic episode	12/46 (26.1%)	11/46 (23.9%)	
Diagnosis: Bipolar disorder	5/46 (10.9%)	4/46 (8.7%)	
Diagnosis: Schizoaffective disorder	3/46 (6.5%)	3/46 (6.5%)	
Drug-naïve/not exposed to antipsychotics	12/46 (26.1%)	14/46 (30.4%)	0.817
Current smoking	19/46 (41.3%)	28/46 (60.9%)	0.095

Note: Continuous variables are shown as mean ± SD or median (IQR), according to distribution. Categorical variables are n/N (%). *p* values are from Mann–Whitney U/Welch *t*-test, χ^2^ test, or Fisher’s exact test, as appropriate. N/A—not Applicable.

**Table 2:** Metabolic and cardiovascular variables.

Variable	Catatonia (*n* = 46)	Control (*n* = 46)	*p* Value
BMI, kg/m^2^	23.4 (22.0–28.0)	22.7 (21.5–29.7)	0.825
Triglycerides	84.5 (65.2–125.2)	98.5 (55.2–110.5)	0.767
Total cholesterol	177.5 (149.0–204.0)	178.0 (152.0–204.8)	0.882
HDL cholesterol	50.4 ± 12.5	48.8 ± 11.5	0.511
LDL cholesterol	107.5 (82.0–130.2)	110.0 (86.0–130.2)	0.673
Diabetes mellitus	5/46 (10.9%)	4/46 (8.7%)	1.000
Arterial hypertension	11/46 (23.9%)	11/46 (23.9%)	1.000

**Table 3:** Complete blood count parameters and CBC-derived inflammatory indices.

Variable	Catatonia (*n* = 46)	Control (*n* = 46)	Raw *p*	Bonferroni-Adjusted *p*
Leukocytes	8.30 ± 2.92	7.09 ± 1.61	0.016	
Neutrophils	4.75 (3.54–7.08)	4.14 (3.15–5.13)	0.033	
Lymphocytes	2.19 (1.44–2.56)	2.05 (1.61–2.35)	0.784	
Monocytes	0.58 (0.42–0.77)	0.52 (0.39–0.60)	0.092	
Platelets	268 (232–292)	236 (200–259)	0.013	
NLR	2.35 (1.60–3.98)	2.08 (1.67–2.50)	0.112	0.560
PLR	123.9 (100.3–190.7)	115.2 (97.3–140.4)	0.116	0.578
MLR	0.27 (0.20–0.38)	0.23 (0.19–0.31)	0.037	0.184
SII	584 (468–823)	476 (339–619)	0.006	0.032
SIRI	1.23 (0.86–2.51)	1.06 (0.62–1.35)	0.020	0.099

Note: CBC and inflammatory indices were compared using Mann–Whitney U or Welch *t*-tests according to distribution. Bonferroni correction was applied only to the five primary inflammatory indices (NLR, PLR, MLR, SII, SIRI). Individual complete blood count parameters were analyzed as exploratory component-level variables, and their reported *p* values are nominal and unadjusted.

**Table 5:** Spearman correlations between inflammatory indices and the number of documented catatonic signs among catatonia cases.

Marker	*n*	Spearman ρ	Raw *p*	Bonferroni-Adjusted *p*
NLR	46	0.212	0.157	0.787
PLR	46	0.066	0.661	1.000
MLR	46	0.227	0.130	0.650
SII	46	0.098	0.518	1.000
SIRI	46	0.184	0.220	1.000

Note: Analyses were restricted to the catatonia group.

**Table 6:** Exploratory ROC analysis for discrimination of catatonia status.

Marker	AUC (95% CI)	Optimal Cut-off	Sensitivity	Specificity	Youden Index	Adj. *p*
NLR	0.596 (0.475–0.712)	4.09	0.261	1.000	0.261	0.560
PLR	0.595 (0.476–0.715)	159.34	0.348	0.957	0.304	0.578
MLR	0.627 (0.511–0.737)	0.45	0.239	0.978	0.217	0.184
SII	0.665 (0.550–0.773)	501.27	0.652	0.652	0.304	0.032
SIRI	0.641 (0.526–0.750)	2.43	0.304	0.957	0.261	0.099

Note: AUC 95% CIs were obtained by bootstrap resampling. Optimal cut-offs were selected by the Youden index and were derived from the same sample in which performance was evaluated, without internal or external validation; they are reported for descriptive purposes only and should not be considered clinically validated thresholds. Adjusted *p* values correspond to Bonferroni-corrected Mann–Whitney comparisons.

## Data Availability

The data presented in this study are available on reasonable request from the corresponding author. The data are not publicly available due to privacy and ethical restrictions.

## Abbreviations

The following abbreviations are used in this manuscript:

AUC	area under the curve
BMI	body mass index
CBC	complete blood count
CI	confidence interval
CRP	C-reactive protein
HDL	high-density lipoprotein
ICD-11	International Classification of Diseases, 11th Revision
IQR	interquartile range
LDL	low-density lipoprotein
MLR	monocyte-to-lymphocyte ratio
NLR	neutrophil-to-lymphocyte ratio
PLR	platelet-to-lymphocyte ratio
ROC	receiver operating characteristic
SD	standard deviation
SII	systemic immune–inflammation index
SIRI	systemic inflammation response index
SPSS	IBM SPSS Statistics
